# Emergence of universal antiretroviral therapy coverage in South Africa: applying the advocacy coalition framework to refine the narratives and inform epidemic responses

**DOI:** 10.11604/pamj.2022.42.6.28141

**Published:** 2022-05-05

**Authors:** Benjamin Momo Kadia, Christian Akem Dimala, Kevin Pene Njefi

**Affiliations:** 1 Health Education and Research Organization, Buea, Cameroon,; 2 Health and Human Development (2HD) Research Network, Douala, Cameroon,; 3 Northwest Regional Delegation for Public Health, Bamenda, Cameroon

**Keywords:** HIV/AIDS, antiretroviral therapy, advocacy coalition

## Abstract

South Africa possesses the largest anti-retroviral therapy (ART) program in the world, but the path to this record was dramatic. There is scarce literature employing a comprehensive framework to explain this achievement and inform epidemic responses. This paper applies the Advocacy Coalition Framework (ACF) to analyse the interactions among diverse actors, institutions and networks that were associated with the AIDS policy change in South Africa. Post-apartheid, HIV/AIDS and AIDS-related mortality were serious public health problems. At the time, the discernible coalitions in the HIV/AIDS policy subsystem were the pro-science coalition and AIDS dissidents. In view of the availability of compelling scientific evidence on the pathogenesis of HIV/AIDS, the clinical usefulness of ART, the availability of funding for national ART roll-out, strong global advocacy to reduce the cost of ART, all of these in an era when access to adequate HIV treatment/care was increasingly considered a human right, the environment to establish an appropriate HIV/AIDS policy for the country was conducive. However, AIDS dissidents dominated the policy agenda via their control over key institutions, the use of various dimensions of power, biasing evidence to inform policy, and promoting the activities of strong interest groups that were not in support of ART. National ART roll-out finally emerged as a political priority because of external shocks (on the AIDS policy subsystem) which disfavoured the dominant coalition. As in this important experience in the history of HIV treatment, stakeholders involved in epidemic response tend to engage in intense ideological conflicts. An adequate appraisal of the outcomes of these conflicts in terms of population health gains and adopted public health and social measures to control epidemics would require the supplementation of complex system thinking with relevant public policy concepts, notably power dimensions, governance, emergence of global health networks and evidence use in policy.

## Essay

The advent of antiretroviral therapy (ART) has greatly revolutionized HIV treatment and care and over the years, tremendous efforts to expand ART coverage have been observed in resource-limited settings [1]. In sub-Saharan Africa, where over two-thirds of persons infected with HIV live, up to 12 million people were reported to be on treatment by 2016 [2]. This corresponded to an estimated double fold increase in ART coverage over the preceding five years [2]. With regard to South Africa, the country with the largest national ART program in the world [3-5], the path to national ART roll-out was extremely dramatic and even linked with the death of hundreds of thousands of persons living with HIV/AIDS [6]. Even though the country's pursuit of national ART roll-out post-apartheid has been extensively described using diverse approaches [7-17], there is scarce literature applying a comprehensive framework to analyse the roles of state and non-state actors who were involved in the process of AIDS policy change. In view of this important gap in the literature and the intense ideological conflicts which characterized the quest for national ART roll-out in South Africa, this paper employs the Advocacy Coalition Framework (ACF) of Sabatier and Jenkins-Smith [18, 19] to analyse and summarize interactions among diverse actors, institutions and networks that were associated with AIDS policy change in South Africa. A Medline search was done using the terms ‘HIV’ OR ‘AIDS’ OR ‘antiretroviral’, AND ‘policy*’ AND ‘South Africa’ and their variations, with search periods from 1981 to 2020 to gather relevant published data on the AIDS policy change process in South Africa. The authors also draw from other relevant public policy concepts to further clarify the policy analysis and use their interpretations to discuss how this landmark experience should inform policymaking in epidemic response.

The ACF proposes that a policy agenda or policy change is the result of competition among coalitions that exist within policy subsystems. A coalition comprises actors, institutions, networks or interest groups that share common fundamental belief systems regarding issues within a subsystem [18, 19]. Belief systems range from the way issues are perceived or framed (deep core beliefs) to solutions (policies) that should be formulated to address the issue (policy core beliefs). According to the ACF, belief systems are scarcely backed by personal interests, but the exact motives for supporting specific coalitions are varied. In the long run, core beliefs tend to remain stable, but changes in secondary aspects of belief systems may occur even in the short run. Nonetheless, a shock from within (internal shock) or out of (external shock) a policy subsystem could potentially lead to adjustments in belief systems or major policy changes in favour of specific coalitions [18, 19]. Based on the ACF, some actors do not belong to any coalition but contribute to the policymaking process by promoting opportunities for coalitions to negotiate and come to compromises. These actors are referred to as policy brokers. Policy-oriented learning, a means by which coalitions improve their understanding of variables which are consistent with their policy cores, continuously occurs within and across coalitions [18, 19].

There were two discernible coalitions in the country's AIDS policy subsystem post-apartheid. AIDS dissidents comprised the minority (yet dominant) coalition. Their deep core beliefs were that, HIV is harmless and does not lead to AIDS. They also believed symptoms of AIDS were consequent to poor nutrition, poverty, and ART. They strongly discouraged ART by focusing on its toxic effects. Their policy core beliefs were that improvements in nutrition with food such as garlic, lemon juice, and beetroots could serve as treatment for HIV/AIDS. Within the country, denialists also promoted scientifically indefensible policies by scaling-up traditional remedies, various chemicals, and other alternative therapies. Members of this coalition included important members of the government like Thabo Mbeki (second post-apartheid president) and some ministers, notably, Tshabalala-Msimang (first post-apartheid minister of health). At the international level, members included renowned scientists (like Peter Duesberg and David Rasnick) [20], anti-AIDS organizations such as the Alive and Well AIDS Alternatives (founded by an HIV positive activist, Christine Maggiore) and actors in the popular media notably, Neville Hodgkinson.

The other coalition comprised advocates of science-based medicine. Their deep core beliefs were that HIV infection is caused by the virus called HIV, which is transmitted via blood and other body fluids and without appropriate treatment HIV would lead to AIDS. Their policy core beliefs were that ART had proven efficacy in the prevention and treatment of HIV and should therefore be prescribed to persons living with HIV/AIDS. They fought for the national roll-out of ART in order to reach out to the thousands of people living with HIV/AIDS and to prevent maternal to child transmission of HIV. The members of this coalition included civil society groups such as Treatment Action Campaign (TAC), some government officials like Jeff Radebe and Madlala-Routledge (from the ministry of health), scientists like William Makgoba (former leader of the Medical Research Council), the media and the South African Medical Association. At the international level, members included scientists, AIDS activists, international associations of persons living with HIV/AIDS, popular media. Several delegates at the 16^th^ international AIDS conference had views that matched with this coalition [21].

The first AIDS-related deaths occurred in 1981 and 1982, but little attention was accorded to the epidemic over the following decade [22]. In 1992, civil society advocated for the creation of the National AIDS Convention, which produced a national AIDS plan. Two years later, the plan was adopted by Nelson Mandela's government [8] and two members of the team that drafted the plan (Dlamini-Zuma and Tshabalala-Msimang) were appointed the first post-apartheid health ministers. This bottom-up approach favoured by Mr Mandela's regime to tackle HIV/AIDS appeared to be effective as more cases were detected, and the prevalence of HIV rose from 0.8% in 1990 to 4.3% in 1994 [22]. Again, such an approach in a unitary state possibly indicated the pro-democracy stance of the then regime.

From the end of 1994 to 1997, a series of events heralded AIDS denialism. First, in 1994, there was a scandal (about a poorly managed HIV/AIDS awareness play called Sarafina II) involving Dlamini-Zuma who was also opposing the introduction of ART for prevention of mother-to-child transmission of HIV. The motives for this minister's change from an advocate of the national AIDS plan to an opponent of ART were unclear. Then, in 1997, a chemical called virodene was being promoted by a university scientist, Olga Visser and Mr Mbeki (then Deputy President), with the latter declaring over media that he felt privileged to have encountered HIV-infected persons who admitted feeling better with virodene. This could only have encouraged more infected persons to take virodene and illustrates how ideological power had been used by Mr Mbeki to favour untested therapies in place of ART. However, the use of virodene was not approved by the South African Medical Control Council which, legitimately, was the drug regulatory authority of the country.

The coalition of AIDS dissidents became more evident in 1999. That year, on becoming the head of state, Mr Mbeki formed an association of AIDS dissidents. He informed the National Council of Provinces that Zidovudine (AZT), an antiretroviral drug, was toxic and he wanted clarifications on the use of the drug. In 2000, he set-up a Presidential AIDS Advisory Panel (comprising orthodox scientists and denialists). With reference to the ACF, it is not clear whether Thabo Mbeki wanted to play the policy broker (which is less plausible since he was clearly an AIDS dissident) or if it was a genuine policy-oriented learning process across coalitions although there were no evident shifts in belief systems. Whichever the scenario, the creation of this panel by the president may have been consequent to Luke's second dimension of power: it was alleged that the creation of the panel was enhanced by advice from foreign dissidents (notably, Peter Duesberg and David Rasnick) [20]. By the end of 2000, after several criticisms by the media, Mr Mbeki withdrew from public commentary on HIV/AIDS and Tshabalala-Msimang managed the AIDS policy agenda. Nonetheless, it would appear he still, though latently, spear-headed decisions on AIDS policy in favour of AIDS dissidents (Luke's second face of power). In 2001, scientific reports revealed significant numbers of AIDS-related deaths. While this could have been regarded as a policy-learning opportunity to the dissidents or an internal shock on the AIDS policy subsystem against the dissidents, the latter rather framed the information to match their core beliefs: Mr Mbeki suggested that the figures had been overestimated and the reports had to be discounted for the nation to focus on other social priorities. Such framing in the face concerning data on the burden of HIV/AIDS may be regarded as an exertion of ideological power with the intention to make the core beliefs of dissidents to prevail at all cost. And even though these reports may have truly been an attempt of technical bias to influence the AIDS policy in favour of science, statistical models had later demonstrated the strong likelihood of several thousands of persons with HIV/AIDS dying as a result of lack of access to ART [6].

In 2003, after a court ruling against Tshabalala-Msimang who had been resisting ART roll-out, the South African cabinet announced its plan to roll out ART in the public health sector. However, in 2005, Dr William Makgoba, an immunologist who was president of the national medical research council (MRC) and an advocate of evidence-based medicine, left the MRC. This could be interpreted as an external shock that disfavoured advocates of science-based medicine (to which Makgoba belonged), as the MRC became less independent. A similar weakening occurred in the Medical Control Council (MCC), but the reason for this is unclear. AIDS dissidents in the government then took advantage of these external shocks to foster technical-bias in evidence as a basis for AIDS policymaking, since the country's relevant regulatory institutions (MRC and MCC) had been compromised. For example, in 2005, government supported a claim that trials on micronutrients produced by Rath Foundation (owned by Matthias Rath, a German entrepreneur) had revealed that high doses of vitamins reversed the course of HIV/AIDS [6]. As a result of this technical bias in the creation of evidence, vitamins were distributed in large quantities in place of ART. Another example which occurred much later was the distribution of an untested concoction called ‘Ubhejane’ via public health facilities. Although it had been reported that this concoction was associated with adverse events like liver failure and the development of resistance to ART, dissidents in the government rather decided to cherry-pick and divulge unfounded information suggesting beneficial effects of ‘Ubhejane’ [6]. Such technical bias in the creation and selection of evidence enabled dissidents to control the AIDS policy to suit their core beliefs.

In November 2005, the Treatment Action Campaign (TAC) and the South African Medical Association (SAMA) jointly filed court papers against the Minister of Health (Tshabalala-Msimang), Matthias Rath and several other AIDS denialists. They were against denialists distributing untested products in the country [23]. After the court ruled in favour of the TAC and SAMA, Tshabalala-Msimang made public declarations suggesting that ART were toxic, adding that she was being forced to give ‘poison to her people’, nutrition was more beneficial, and patients had the right to choose their treatment strategies. These assertions led to confusion among persons living with HIV/AIDS and there was widespread drop of ART for scientifically indefensible remedies. As was previously illustrated, such use of power as thought-control mechanism seemed to be an important strategy used by dissidents to sustain their policy core beliefs.

The Toronto International AIDS Conference of 2006 hugely condemned Mr Mbeki and Tshabalala-Msimang over the country's AIDS policy. This, among others, prompted revolts in the African National Congress (the ruling party in South Africa since the end of apartheid) and the cabinet of South Africa [6]. Subsequently, the cabinet reasserted itself over presidential authority by transferring responsibility for the AIDS policy to Deputy President Mlambo-Ngcuka. This series of shocks which disfavoured AIDS dissidents was not yet over: Tshabalala-Msimang took a sick leave later in the year 2006 and the deputy health minister, Madlala-Routledge, together with interim minister of health, Jeff Radebe, who were advocates of science-based medicine took over the AIDS policy agenda. They started working with the civil society and health professionals and set the objectives of halving HIV infections and expanding ART coverage to 80% by 2011. They also planned to restructure the National AIDS Council. However, this window of opportunity (as Kingdon's model of policymaking [24, 25] would refer to regarding the advocacy for national ART roll-out by advocates of science-based medicine) was shut when Tshabalala-Msimang returned from leave in 2007 and restarted countering national ART roll-out. She side-lined Madlala-Routledge who was ultimately fired by President Mbeki. Reports also indicate that in the same year, Tshabalala-Msimang started formulating new legislations to regulate use of alternative medicine among persons living with HIV/AIDS [6]. However, in 2008, there were major changes in government, and these appeared to be the greatest external shocks which favoured the coalition of science advocates: Mr Mbeki's rule ended, and his successor removed Tshabalala-Msimang from office. Universal ART coverage then became a top priority on the AIDS policy agenda. This, in unison with the pre-existing national and international efforts to scale-up ART use in the country led to the emergence of national ART roll-out in the republic of South Africa.

Knowledge of the actors, context and processes involved in the AIDS policymaking is key to understanding the direction of policy change. Nonetheless, historical analyses highlighting the variety of instruments that could be employed by the actors is also fundamental to elucidate the drivers of policy change. The ACF is generally applied to domestic contexts, as its initial construct was designed to understand how different actors worked together through the policy process to implement change in the United States environmental policy [26]. In this paper, we expand the application of the framework to the transnational level by capturing various actors, networks, and institutions at the international scene that actively fostered core beliefs of coalitions. It is worth mentioning, in passing, that Sabatier *et al*. had long encouraged the application of the framework on a wider scope [19] even though this is still lacking in current literature. The paper is an important attempt to enlighten the public and policymakers on the variety of instruments and processes that can be employed in the arena of policymaking in epidemic response. It also serves to inform policymakers on the merits and demerits of employing certain policy instruments within a given context and how the interplay between governance and the use of these instruments could impact population health in epidemics. Importantly, the transnational approach used in this paper makes our analysis highly relevant in an era characterized by pandemics such as COVID-19 outbreak and Ebola outbreaks.

Post-apartheid and for over a decade, high rates of HIV/AIDS and AIDS-related mortality constituted serious public health menaces in South Africa, even though there was a conducive environment to develop an appropriate HIV/AIDS policy. This conducive environment was due to the availability of compelling scientific evidence on the pathogenesis of HIV/AIDS and the benefits of ART, availability of funding for national ART roll-out, strong global advocacy to reduce the cost of ART [27], all of these being present in an era when inaction against HIV/AIDS was literally considered a crime [11, 28-30]. However, dissidents who constituted the minority coalition, dominated the HIV/AIDS policy agenda via their control over key national institutions, the use of various dimensions of power, biasing evidence to inform policy, and promoting the activities of strong interest groups notably, Traditional Healers' Organization, businessmen and charlatans. National ART roll-out ultimately emerged as a political priority because of external shocks (changes in leadership and governance) which disfavoured the dissidents' coalition.

From another perspective, the response to the public health menace posed by HIV/AIDS in South Africa could be regarded as the result of the way the menace was framed by diverse actors and institutions, especially those that had access to some form of power. Two important examples are worth citing. First, it was suggested that Mr Mbeki framed the menace and the proposition of rolling-out ART as the products of conspiracies spearheaded by Western societies and he therefore countered every effort to roll out ART in his high authority as president [29]. In line with the ACF, this may have been the origin of his core belief as an AIDS dissident and slowed down national ART roll-out. In a like manner, conspiracy beliefs have greatly shaped health belief models and health seeking behaviours in the COVID-19 global outbreak. This is reflected in the considerable vaccine hesitancy rates worldwide and the negative perceptions about the COVID-19 vaccine in Africa. The second example relates to the Sarafina play. Albeit the second part of the Sarafina play received several criticisms, it can be argued that the production of such a play illustrates that its initiators had framed HIV/AIDS in South Africa as an issue requiring the use of complex biosocial interventions and not just ART for the effective control of the pandemic. The importance of behavioural change as part of public health and social measures to control more recent pandemics like COVID-19 cannot be overemphasized.

Even though the ACF is one of the most comprehensive frameworks for explaining policy change, failure to borrow from other public policy concepts such as governance and policy implementation, dimensions of power, framing of global health issues, evidence use in policy and emergence of global health networks to supplement the ACF would lead to suboptimal appraisal of the AIDS policy change and misinformation of policies adopted to response to infectious disease epidemics. Such complex system systems thinking approach should not only serve to better understand policymaking processes, but should also provide insights into the potential downstream effects of adopted policies and strategies to appropriately fine-tune them for population health gains. Kapiriri *et al*. conducted a comprehensive literature review to comparatively assess the response to the SARS, Zika and Ebola outbreaks. Although they did not apply a comprehensive framework such as the ACF to analyse the available evidence, the themes that emerged from the review revealed that epidemic response tends to be political, public health measures to control epidemics are not necessarily informed by credible evidence (i.e. evidence could be biased at different levels), response strategies tend to be determined by actors or institutions that have some form of power [30]. These observations largely concur with the key findings.

The main limitation of this paper is that the historical analysis was not supplemented with evidence from key informant interviews and other qualitative data collection methods. This could have provided more perspectives (including those from civil society, persons living with HIV/AIDS and other stakeholders) on the policy change process and how the AIDS policy change was embraced by key stakeholders involved in the management of the pandemic.
